# The need to redefine age- and gender-specific overweight and obese body mass index cutoff points

**DOI:** 10.1038/nutd.2015.36

**Published:** 2015-11-30

**Authors:** A M Nevill, G S Metsios

**Affiliations:** 1 Faculty of Education, Health and Wellbeing, University of Wolverhampton, Walsall, UK; 2 Research Institute in Physical Performance and Rehabilitation, Centre for Research and Technology, Thessaly, Trikala, Greece; 3 Department of Rheumatology, Dudley Group of Hospitals NHS Trust, Russell's Hall Hospital, Dudley, West Midlands, UK

## Abstract

For convenience, health practitioners and clinicians are inclined to classify people/patients as overweight or obese based on body mass index (BMI) cutoff points of 25 and 30 kg m^−2^ respectively, irrespective of age and gender. The purpose of the current study was to identity whether, for the same levels of adiposity, BMI is the same across different age groups and gender. A two-way ANCOVA revealed significant differences in BMI between different age groups and gender (plus an interaction), using body fat (%) as the covariate, data taken from a random sample of the English population (*n*=2993). Younger people had greater BMI than older people for the same levels of adiposity (differences ranged by 4 BMI units for males, and 3 BMI units for females). In conclusion, if BMI thresholds for overweight (BMI=25 kg m^−2^) and obese (BMI=30 kg m^−2^) are to reflect the same levels of adiposity across all gender and age groups within a population, then age- and gender-specific BMI adjustments outlined here are necessary to more accurately/fairly reflect the same critical levels of adiposity.

## Introduction

Body mass index (BMI) is undoubtedly the most frequently used proxy of adiposity/obesity in large epidemiological studies in both healthy and diseased populations. Despite its wide use, which pertains to convenience since it only requires the measurement of height and mass, BMI has been frequently criticised as having various deficiencies as a measure of obesity^1^ both for healthy and diseased populations.^2, 3^

One of the major issues with BMI is that it does not reflect the changes in body composition that occur with age, in particular the presence of sarcopaenia, which is characterised by reduced muscle mass and increased adiposity. As such the utilisation of BMI in evidence-based approaches relevant to dietary interventions and/or clinical decision making needs to be reconsidered and where appropriate, readjusted. Therefore, the aim of the present study was to investigate the cutoff points of BMI in relation to adiposity in a large cohort of participants in order to validate if the established cutoff points accurately reflect adiposity.

## Patients and methods

The current data, used to explore the association between BMI (kg m^−2^) and body fat percentage (BF%), has been previously published^4^ although originally obtained from the Allied Dunbar National Fitness Survey (ADNFS) (1992). The ADNFS recruited 4316 randomly selected healthy participants, aged 16 years and over, from thirty English parliamentary constituencies. A sub-sample took part in a physical appraisal yielding BMI and estimates of BF% data for 2993 healthy people (male *n*=1420; female *n*=1573). Estimates of BF%, taken for the ADNFS, were determined using the methods based on skin fold thicknesses at four sites; the biceps, triceps, sub-scapular and supra-iliac.^5^

### Statistical methods

In order to detect any systematic differences in gender and age groups (16–29, 30–39, 40–49, 50–59, 60–69, 70–79, 80+, age in years) holding BF% constant, a two-way (gender-by-age group) analysis of covariance (ANCOVA) was employed using BF% as the covariate. Finally, we have used Bonferroni multiple comparisons to investigate BMI differences amongst the different age groups. The level of significance was set at *P*<0.05 and all the analyses were conducted with the Statistical Package for the Social Sciences (SPSS) version 20.

## Results

**Figure 1:**
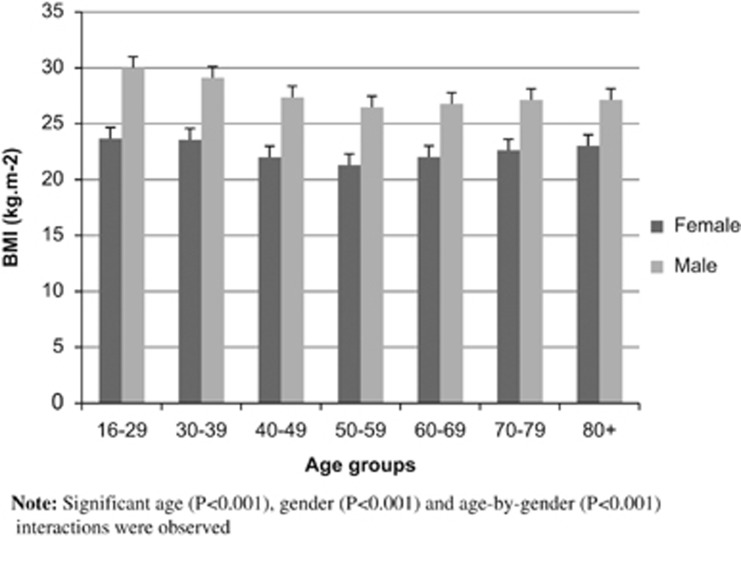
Mean (±s.e) BMI by age group and by gender holding BF% constant, all BMIs evaluated at BF%=29.49%.

The relationship between BMI and BF% was found to be approximately linear. The ANCOVA revealed significant main effects of age group (*P*<0.001) and gender (*P*<0.001), and a significant age group-by-gender interaction (*P*<0.001) together with a significant covariate of BF% with the BMI slope parameter B=0.570 (s.e.=0.010) per unit BF%, means (±s.e.) given in Figure 1. The group-by-gender interaction was due to a greater difference in BMI between the younger and older males compared with the difference in BMI observed in females.

The median age of the sample was 45 years. As such, taking the ‘anchored' baseline group as the 40- to 49-year-old group, and assuming the overweight and obese threshold for these groups are BMI=25 and 30 kg m^−2^, respectively, we estimated the BMI of all other age groups and by gender, based on the differences observed in Figure 1. Anchoring the overweight threshold BMI value=25 kg m^−2^ for the 40–49 years age group, all other age group and gender differences are estimated in Table 1 (BMI means (rounded to a whole BMI unit number) estimated for the same BF%=25.3 and 34.8 for all male and female participants, respectively). Similarly anchoring the obese threshold BMI value=30 kg m^−2^ for the 40–49 years age group, all other age group and gender differences are estimated in Table 1 (BMI means (rounded) estimated for the same BF%=34.1 and 43.5 for all male and female participants, respectively).

## Discussion

For the same level of adiposity (using BF%), systematic differences in BMI were found in different gender and age groups from a randomly selected national sample (male *n*=1420; female *n*=1573) taken from 30 English parliamentary constituencies (Figure 1). On the basis of these differences, the BMI means (rounded), calculated and reported in Table 1 suggest that younger males and, to a lesser extent female participants have significantly higher levels of BMI compared with their older counterparts, for the same levels of adiposity (BF%). This is unsurprising given that younger males are likely to be more active than younger females and that physical activity will naturally decline in both genders in older people. This trend is well documented with ageing,^6^ in particular the presence of sarcopaenia that is characterised by reduced muscle mass and increased adiposity, the latter being the result of lower energy expenditure.

Although this trend is well known amongst health practitioners, most still persist in prescribing common BMI thresholds for being overweight (BMI=25 kg m^−2^) and obese (BMI=30 kg m^−2^) irrespective of the individuals age and gender. Our findings suggest an alternative strategy should be considered. Given that younger males will have a higher percentage of muscle mass than a 40- to 49-year-old male, their BMI obesity threshold could be raised to a less restrictive 33 kg m^−2^ (as per our relevant calculations in Table 1) to equate to the same level of adiposity as their older 40- to 49-year-old counterparts. A similar recommendation can be made for younger females, that is, their BMI obesity threshold could be raised to 32 kg m^−2^ (see Table 1 for newly developed cutoff points) to equate to the same level of adiposity as a 40- to 49-year-old female. In contrast, the BMI obesity threshold for both male and female 50- to 59-year-old participants could be reduced to a more restrictive/conservative level. For these older age groups, we would recommend that such participants are regarded as obese once their BMI exceeds 29 rather than 30 kg m^−2^ (to equate with the same level of adiposity associated with 40- to 49-year-old subjects). Similar adjustments would be required for the overweight thresholds for the various gender and age groups outlined in Table 1.

In conclusion, if BMI thresholds for overweight and obese participants are to reflect similar levels of adiposity across all gender and age groups within a population, then age- and gender-specific BMI adjustments outlined in Table 1 are necessary to more accurately and fairly reflect the same levels of adiposity.

## Figures and Tables

**Table 1:** Redefined overweight and obese BMI thresholds (rounded) for different gender and age groups

	*Overweight*		*Obese*	
	*Male*	*Female*	*Male*	*Female*
16–29	28^b^	27^b^	33^b^	32^b^
30–39	27^b^	27^b^	32^b^	32^b^
40–49^a^	25	25	30	30
50–59	24^b^	24^b^	29^b^	29^b^
60–69	24	25	29	30
70–79	25	26	30	31
80+	25	26	30	31

Abbreviation: BF%, body fat percentage; BMI, body mass index.

Overweight BMI means (rounded) evaluated at BF%=25.3 (male) and 34.8 (female).

Obese BMI means (rounded) evaluated at BF%=34.1 (male) and 43.5 (female).

Bonferroni multiple comparisons: significantly different from the anchored age group.

a Taking the age group 40–49 years as the anchored baseline group; BMI=25 and 30 kg m^−2^, respectively.

b *P*<0.001.
